# 472. Clinical Outcomes of Solid Organ Transplant Recipients with Severe Acute Respiratory Syndrome Coronavirus 2: 2021 Update

**DOI:** 10.1093/ofid/ofab466.671

**Published:** 2021-12-04

**Authors:** Deeksha Jandhyala, Jessica Lewis, Ruth Adekunle

**Affiliations:** Medical University of South Carolina, Charleston, South Carolina

## Abstract

**Background:**

The Centers for Disease Control and Prevention identified solid organ transplant (SOT) recipients as persons at high risk to develop severe illness secondary to Severe Acute Respiratory Syndrome Coronavirus 2 (SARS-CoV-2). We reviewed the clinical characteristics and outcomes of SOT recipients who had SARS-CoV-2 at our center.

**Methods:**

This was a retrospective review of SOT recipients diagnosed with SARS-CoV-2 between March 1^st^ 2020 and February 28^th^ 2021 in the Medical University of South Carolina (MUSC) Health Care system. Subjects were included if they had undergone SOT at any time prior to a positive SARS-CoV-2 PCR. Descriptive statistics were used to analyze demographic and clinical characteristics. Primary outcomes included need for hospitalization, complications, and estimated all-cause mortality.

**Results:**

96 SOT recipients were diagnosed with SARS-CoV-2. Seven patients were excluded as they initially tested positive and were admitted to another facility or had a simultaneously positive IgG. 89 SOT recipients were analyzed, of which, 39 (44%) were managed as an outpatient and 50 (56%) required admission. 13 (33%) of outpatient recipients were treated with a monoclonal antibody. Of those hospitalized, 24 (48%) were male, 34 (68%) were Black, and 34 (68%) were kidney transplant recipients. Median age at positive test was 53 years of age and median time from transplant to positive test was 3.1 years. Median length of stay was three days (range 0.1–108.6). 17 (34%) patients required supplemental oxygen and 11 (22%) received care in the intensive care unit. Complications, including bacterial infections (16%), fungal infections (2%), CMV reactivation (6%), and rejection (2%) were rare. Three patients (3%) died during the study time period.

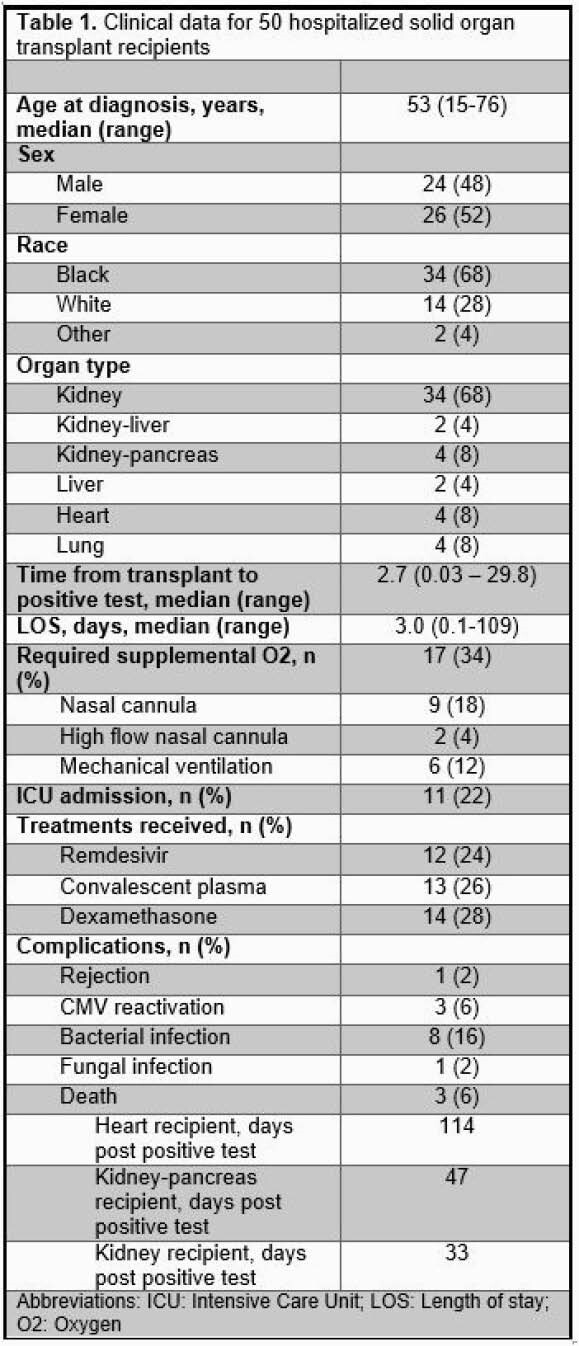

**Conclusion:**

The majority of SOT recipients with SARS-CoV-2 in our cohort required admission, however they experienced few complications and a low mortality, despite their high-risk status.

**Disclosures:**

**All Authors**: No reported disclosures

